# Global Change Reshapes Northern Lakes Towards Browner, More Nutrient‐Depleted and Nitrogen‐Limited Conditions With Contrasting Impacts on Phytoplankton Biomass

**DOI:** 10.1111/gcb.71008

**Published:** 2026-07-22

**Authors:** Ann‐Kristin Bergström, Aleksey Paltsev, Dag O. Hessen, Pirkko Kortelainen, Jussi Vuorenmaa, Heleen A. de Wit, Danny C. P. Lau, Tobias Vrede, Kristiina Vuorio, Peter D. F. Isles, Anders Jonsson, Erik Geibrink, Kimmo K. Kahilainen, Stina Drakare, Irena F. Creed

**Affiliations:** ^1^ Department of Ecology, Environment and Geoscience Umeå University Umeå Sweden; ^2^ Centre of Biogeochemistry in the Anthropocene and Department of Bioscience University of Oslo Oslo Norway; ^3^ Finnish Environment Institute (SYKE) Helsinki Finland; ^4^ Norwegian Institute for Water Research Oslo Norway; ^5^ Department of Aquatic Sciences and Assessment Swedish University of Agricultural Sciences Uppsala Sweden; ^6^ Watershed Management Division Vermont Department of Environmental Conservation Montpelier Vermont USA; ^7^ Lammi Biological Station University of Helsinki Helsinki Finland; ^8^ Department of Physical and Environmental Sciences University of Toronto Toronto Ontario Canada

**Keywords:** browning, carbon–nutrient stoichiometry, climate change, dissolved organic carbon, nutrient limitation, recovery from acidification

## Abstract

Global change is altering trade‐offs between light and nutrient availability in northern lakes, with implications for biogeochemical cycling and ecosystem functioning. Using 29 years (1991–2019) of long‐term monitoring from 169 Fennoscandian natural lakes, we quantified spatiotemporal change in browning (total organic carbon, TOC), dissolved inorganic nitrogen (DIN), total phosphorus (TP), and stoichiometry (TOC:TP and DIN:TP). We then related these trajectories to co‐occurring trends in air temperature, precipitation, and atmospheric nitrogen (N) and sulfur (S) deposition to evaluate how climate change and deposition recovery jointly reconfigure lake chemistry across subregions. TOC increased widely and DIN declined across most lakes, whereas TP trends were mixed, producing a pervasive rise in TOC:TP and decline in DIN:TP over time. Mixed linear models indicated that TOC increases were most often associated with declining S deposition and increasing precipitation, while DIN was generally positively related to N deposition, with additional subregional roles for temperature and precipitation. As browning coincided with declining DIN:TP, lakes shifted toward darker conditions with greater prevalence of nitrogen and phosphorus co‐limitation and nitrogen limitation relative to phosphorus limitation. In a Swedish subset of 74 lakes, chlorophyll‐*a* trends were heterogeneous among subregions, indicating context‐dependent biomass responses rather than a uniform phytoplankton signal. Together, these results show that ongoing climate change and reduced atmospheric deposition are reshaping carbon–nutrient coupling and nutrient‐limitation regimes across northern lakes, with consequences for future primary production and energy transfer to higher trophic levels.

## Graphical abstract

Fennoscandian lakes have become browner and more nutrient‐depleted, with rising total organic carbon (TOC), declining dissolved inorganic nitrogen (DIN), and, in some regions, declining total phosphorus (TP) concentrations. These changes have increased TOC:TP and lowered DIN:TP, shifts being linked to broad‐scale atmospheric drivers, with regionally distinctive responses in phytoplankton biomass.

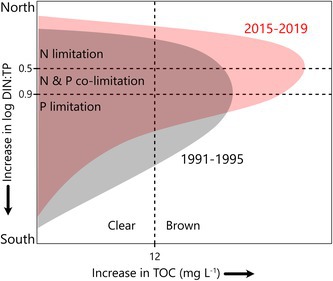

## 1 Introduction

Global change is increasing concentrations of colored dissolved organic matter in many northern lakes, a phenomenon widely termed *browning* and commonly tracked as rising dissolved organic carbon (DOC) (Monteith et al. 2007; de Wit et al. 2016, 2023). Browning is now well documented, and its broad drivers are increasingly understood, yet its interactions with key nutrients—nitrogen (N) and phosphorus (P)—and the ecosystem consequences of these coupled changes remain poorly resolved. Despite the expectation that increased terrestrial organic matter export could elevate total nitrogen (TN) and total phosphorus (TP), several long‐term studies from reference lakes of limited human impact report declining concentrations of dissolved inorganic nitrogen (DIN) and, in some regions, declining TP (Canham et al. 2012; Isles et al. 2018, 2023). These divergent trajectories in DOC, DIN, and TP imply not only changing absolute concentrations, but also shifting stoichiometric balances (e.g., DOC:TP and DIN:TP), with potential consequences for phytoplankton biomass and nutrient limitation regimes (Bergström 2010; Isles et al. 2021; Stetler et al. 2021).

Browning in northern lakes has been observed for decades and was initially attributed largely to recovery from atmospheric sulfur (S) deposition and associated changes in catchment and soil processes (Monteith et al. 2007; Erlandsson et al. 2008). As atmospheric S deposition has declined to relatively low levels, browning is increasingly linked to climate‐driven and hydrological mechanisms, including enhanced forest growth under warming (Freeman et al. 2001; Finstad et al. 2016; Lucas et al. 2016) and/or increased precipitation that promotes DOC mobilization and transport to surface waters (Tranvik and Jansson 2002; de Wit et al. 2016; Imtiazy et al. 2025; Paltsev et al. 2025). Over similar periods, decreasing atmospheric N deposition and related changes in catchment processing have been associated with declining lake DIN and, in some systems, TP (Eimers et al. 2009; Canham et al. 2012; Garmo et al. 2014; Huser et al. 2018; Isles et al. 2018). Browning may partly mitigate nutrient declines, particularly for TP, through increased terrestrial export of organic P, but the strength and consistency of this coupling appear to vary among regions and through time (Kortelainen et al. 2006; Isles et al. 2018). Together, spatial and temporal differences in climate and atmospheric deposition can therefore push lakes toward new combinations of DOC, DIN, and TP concentrations, altering DOC:TP and DIN:TP stoichiometry and potentially shifting lake productivity and nutrient limitation (Hessen et al. 2009; Isles et al. 2021; Bergström et al. 2022). However, the extent to which atmospheric drivers coherently explain long‐term trends in lake water chemistry, and whether those relationships are consistent across space and time, remains insufficiently tested, often because long and harmonized datasets spanning broad environmental gradients have been limited.

The rise in DOC can influence phytoplankton through two opposing ways: DOC reduces light availability (negative effect) while terrestrial inputs may increase nutrient availability (positive effect) (Kelly et al. 2018; Bergström and Karlsson 2019; Vasconcelos et al. 2019; Isles et al. 2021). These counteracting mechanisms underpin a commonly observed unimodal relationship between DOC and phytoplankton biomass, where biomass increases with DOC at low concentrations but declines once light limitation dominates (Thrane et al. 2014; Kelly et al. 2018; Bergström and Karlsson 2019). Empirically, this transition often occurs at around 10–12 mg L^−1^ DOC (Solomon et al. 2015; Bergström and Karlsson 2019), although thresholds may vary with lake characteristics.

Phytoplankton responses also depend on nutrient supply ratios, particularly DIN:TP, which determine nutrient limitation regimes (Bergström 2010; Elser et al. 2009; Isles et al. 2020). For example, declining DIN:TP and shifts from P‐limitation toward N‐limitation, even without strong DOC change, could reduce phytoplankton biomass via diminished N fertilization linked to declining atmospheric N deposition (Bergström and Jansson 2006; Elser et al. 2009; Bergström et al. 2022). These expectations imply that browning and nutrient trajectories should generate heterogeneous phytoplankton responses across landscapes that differ in catchment properties, climate, and histories of S and N deposition (Hessen 2013; Isles et al. 2021; Johnson et al. 2025).

Despite these conceptual expectations, many assessments of DOC–nutrient–phytoplankton coupling rely on temporally or spatially constrained surveys that may not capture long‐term co‐trends in DOC, DIN, TP, and their stoichiometry (Isles et al. 2021; Stetler et al. 2021; Paltsev et al. 2024). Consequently, comprehensive spatiotemporal analyses that link atmospheric drivers to multi‐decadal water‐chemistry trajectories and then connect those trajectories to phytoplankton biomass and nutrient limitation remain scarce.

Long‐term monitoring programs in Fennoscandia provide a rare opportunity to address these gaps, spanning decades and encompassing lakes across strong gradients in DOC, nutrient status, climate, and atmospheric deposition (Isles et al. 2018, 2021; Paltsev et al. 2024). Here, we use 29 years of data (1991–2019) from 169 lakes to quantify long‐term trends in browning (using total organic carbon, TOC, as a proxy for DOC), DIN, and TP, and to evaluate concurrent changes in carbon‐per‐nutrient availability (TOC:TP) and phytoplankton nutrient supply ratios (DIN:TP). To better capture changes in the optical character of organic matter relevant to light climate, we also examine the specific visible light–absorbing capacity of organic matter using Abs_420_:TOC (absorbance at 420 nm normalized by TOC). We then relate these water‐chemistry trends to trajectories in key atmospheric drivers (air temperature, precipitation, and atmospheric S and N deposition). Finally, using chlorophyll‐*a* (Chl‐*a*) from a subset of Swedish lakes as a proxy for phytoplankton biomass, we assess whether observed changes in water chemistry correspond to changes in phytoplankton biomass and whether shifts in DIN:TP suggest transitions among nutrient limitation regimes.

Specifically, we ask:

1. Are lakes becoming browner and more nutrient‐depleted? How have TOC, DIN, and TP changed over 1991–2019, and what are the resulting trends in TOC:TP and DIN:TP?
2. Are water‐chemistry trends linked to atmospheric drivers? To what extent are trends in TOC, DIN, TP, TOC:TP, and DIN:TP associated with trends in temperature, precipitation, and S and N deposition, and do these relationships differ among Fennoscandian subregions?
3. Do phytoplankton biomass track changes in water chemistry? Are trends in Chl‐*a* consistent with inferred changes in light climate with browning and in nutrient availability?
4. Have nutrient limitation regimes shifted? Do trends in DIN:TP indicate movement toward N–P co‐limitation or stronger N‐limitation, and how do these shifts relate to observed trends in Chl‐*a*?

## 2 Materials and Methods

### 2.1 Meteorological Data

Estimates of air temperature and precipitation were extracted from the TerraClimate dataset, which provides monthly interpolated climate grids at high spatial resolution (1°/24°, ~4 km) (Abatzoglou et al. 2018). For each lake and year (1991–2019), we calculated mean annual maximum air temperature (MAT; °C) and mean annual precipitation (MAP; mm). To characterize long‐term changes, we also calculated 5‐year means of MAT and MAP for each lake for 1991–1995 and 2015–2019.

### 2.2 Atmospheric Deposition

Gridded, interpolated deposition estimates were downloaded from the European Monitoring and Evaluation Programme (EMEP) as NetCDF files for each calendar year from 1991 to 2019 (Colette et al. 2016; Amann et al. 2018). For each lake‐year, we extracted deposition values from the grid cell containing the monitored site. We calculated annual wet inorganic nitrogen deposition (Ndep) as the sum of wet NH_4_^+^ and wet total nitrogen oxides (NO_*x*_ total) deposition, and annual wet sulfur deposition (Sdep) as oxidized sulfur deposition (wet; SO_2_ + SO_4_^−2^). We also calculated 5‐year mean Ndep and Sdep for the first 5 years (1991–1995) and the last 5 years (2015–2019) for each lake.

### 2.3 Lake Monitoring Data, Screening, and Harmonization

We compiled long‐term lake water chemistry data from national monitoring programs in Norway, Sweden, and Finland, focusing on lakes in relatively pristine boreal catchments with minimal direct human disturbance. Sampling design differed among countries: Norway (78 lakes) was sampled annually in autumn (late September–early November), while Finland (26 lakes) and Sweden (97 lakes) were sampled 4–6 times per year. To harmonize across datasets, we used surface‐layer autumn samples only to generate one annual value per lake for 1991–2019 (29 years). Not all lakes were sampled in all years, resulting in temporal gaps. To minimize the influence of missing data on temporal analyses, we retained lakes with ≤ 15% missing annual values across the study period.

**FIGURE 1:**
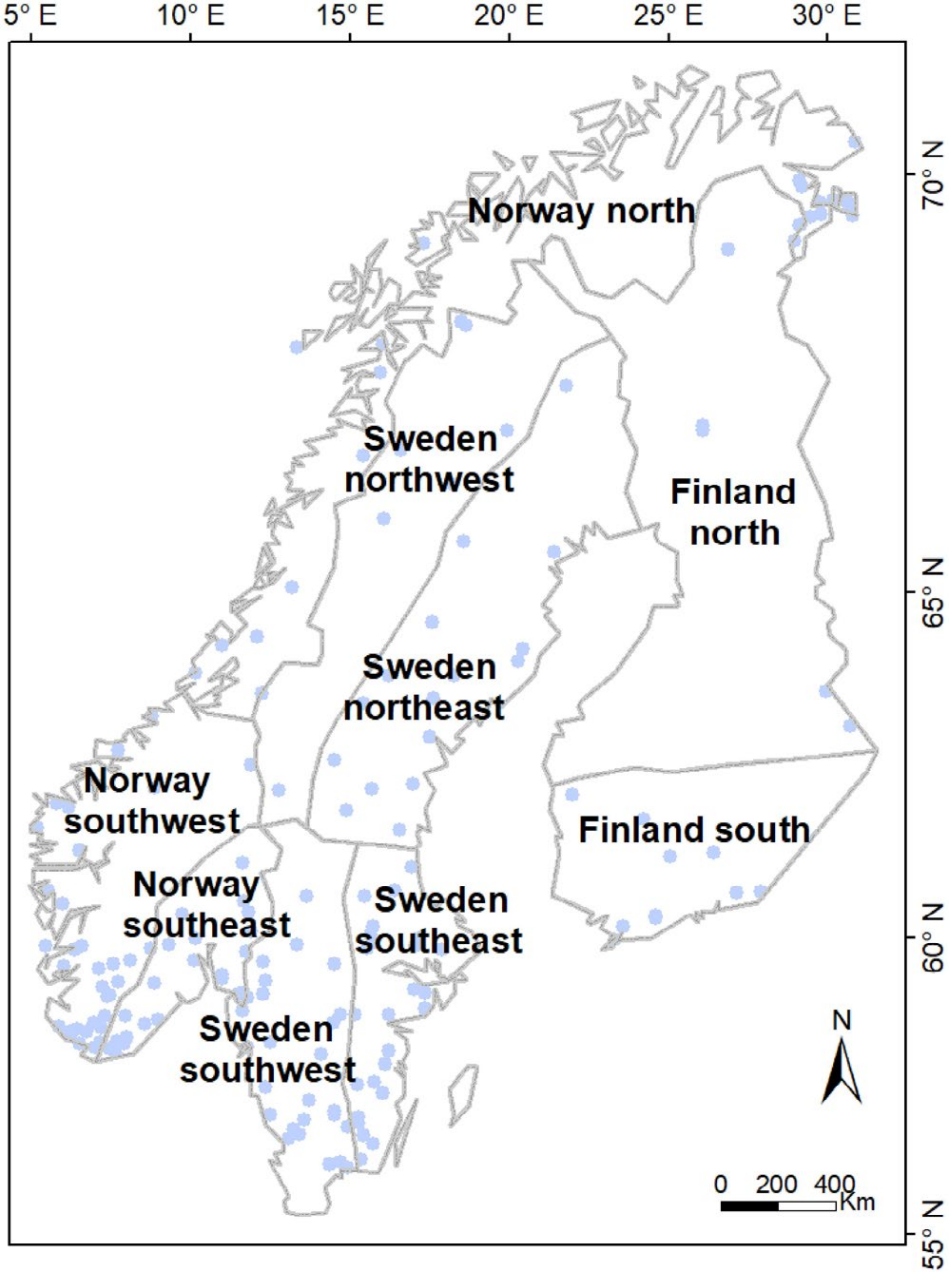
The Fennoscandian subregions in the study.

This screening resulted in 169 lakes: Norway (75 lakes; median and mean ± SD area: 0.52 and 0.79 ± 0.70 km^2^), Finland (19 lakes; 0.31 and 0.53 ± 0.59 km^2^), and Sweden (75 lakes; 0.53 and 1.15 ± 1.90 km^2^). The lakes span a large geographic area encompassing substantial regional differences in climate, atmospheric deposition history, and catchment characteristics, with some variabilities exhibiting contrasting temporal trends among regions. Consequently, a pooled analysis would mask important regional drivers, trends, and responses. We therefore grouped lakes into nine subregions (Figure 1) based on similarities in climate, atmospheric deposition, and biogeographical conditions following the rationale in de Wit et al. (2023). This yielded the following regions: Norway southwest (24 lakes), Norway southeast (13), Norway north (38), Sweden southwest (30), Sweden southeast (15), Sweden northwest (15), Sweden northeast (15), Finland south (10), and Finland north (9). Historically, southern Fennoscandia has received the highest atmospheric S and N deposition, whereas TOC and TP concentrations typically increase from west to east (low levels in Norway to higher levels in Finland) (Henriksen et al. 1998).

All chemical variables were analyzed at accredited laboratories using international (ISO) or European (EN) standards: the Norwegian Institute for Water Research (Skjelkvåle et al. 2001), the Finnish Environmental Administration (1991–2015) and Eurofins Environment Testing Finland (2016–2019) (Vuorenmaa et al. 2014), and the Swedish University of Agricultural Sciences (Fölster et al. 2014), consistent with prior synthesis work (Fölster et al. 2014; Vuorenmaa et al. 2014; de Wit et al. 2023). Dissolved inorganic nitrogen (DIN) was calculated as the sum of NH_4_^+^ + NO_3_^−^. TOC was used as a proxy for DOC because > 90% of TOC is DOC in Finnish and Swedish lakes (Köhler et al. 2002; Kortelainen et al. 2006). For Swedish lakes, absorbance at 420 nm measured in a 5‐cm cuvette (Abs_420_; m^−1^) was available and used to compute the specific visible light‐absorbing capacity of organic matter as Abs_420_:TOC (Abs_420_ [m^−1^]/TOC [mg L^−1^]) (Eklöf et al. 2021; Isles et al. 2021). We calculated DIN:TP (molar ratio), TOC:TP (mass ratio; TOC [μg L^−1^]/TP [μg L^−1^]), and Abs_420_:TOC, and log10‐transformed DIN:TP, TOC:TP, and Abs_420_:TOC prior to analysis (Isles 2020; Isles et al. 2021). All log10‐transformed data is written as log *xx*; for example, log DIN:TP.

In Norway, NH_4_^+^ and TP were not analyzed in some lakes or were analyzed sporadically during the early part of the time series. We therefore calculated lake‐specific mean NH_4_^+^ and TP concentrations for 2002–2019 and used these means to fill missing NH_4_^+^ values and to fill TP for the first 5 years (1991–1995) (and sporadic missing years thereafter). These gap‐filled values were used for constructing DIN and derived ratios in period‐based comparisons; TP trend analyses for Norwegian lakes were restricted to years with measured TP data (see Section 2.4). Across Norwegian lakes, NH_4_^+^ represented a smaller fraction of DIN than NO_3_^−^ (across all lakes and years, mean and median NH_4_ fractions were 29% and 23%, respectively; coefficient of variation 76%), and TP concentrations were generally low; consequently, variation in Norwegian DIN:TP was driven primarily by changes in NO_3_^−^.

### 2.4 Trends in Atmospheric Variables and Lake Water Chemistry

We tested for monotonic trends in atmospheric variables (MAT, MAP, Ndep, Sdep) for each lake using Mann–Kendall tests (Kendall 1975) and estimated rates of change (year^−1^) using Sen's slope (Sen 1968). To summarize regional patterns, we calculated the mean Sen's slope for each subregion as the average of lake‐level slopes (including both positive and negative slopes). We tested whether mean subregional slopes differed from zero using a one‐sample *t*‐test (*α* = 0.05) and report results at *α* = 0.10 to facilitate comparison with previous studies (Bergström et al. 2024; Paltsev et al. 2024). We also averaged subregional mean slopes to describe overall trends across Fennoscandia.

To quantify long‐term changes in water chemistry and stoichiometry, we calculated 5‐year mean values for each lake for 1991–1995 and 2015–2019 for TOC, DIN, TP, log DIN:TP, log TOC:TP, and log Abs_420_:TOC, and expressed changes as relative differences between periods. We tested whether variables differed between the two periods using paired *t*‐tests or, when assumptions were not met, Wilcoxon signed‐rank tests.

To assess whether relationships between TOC and nutrient status or optical properties shifted over time, we regressed each lake's 5‐year mean TP, log DIN:TP, log TOC:TP, and log Abs_420_:TOC against TOC for the two periods (1991–1995 and 2015–2019) and used ANCOVA to test whether regression slopes differed between periods.

In addition to period comparisons, we calculated lake‐specific Mann–Kendall trends and Sen's slopes for TOC, DIN, TP, log DIN:TP, log TOC:TP, and log Abs_420_:TOC. Because Norwegian TP data were largely unavailable prior to 2002, TP trends for Norway were calculated for 2002–2019, whereas all other water chemistry parameters (and all other countries) used 1991–2019. As above, to summarize regional patterns for these parameters, we calculated the mean Sen's slope for each subregion as the average of lake‐level slopes (including both positive and negative slopes). We then tested whether mean subregional slopes differed from zero using a one‐sample *t*‐test (*α* = 0.05) reporting results at *α* = 0.10.

### 2.5 Effects of Atmospheric Drivers on Lake Water Chemistry

We assessed how spatiotemporal variability in atmospheric drivers influenced lake water chemistry within each subregion using mixed linear models (MLM). For each lake‐year, atmospheric variables were represented by MAT, MAP, Ndep, and Sdep, and water chemistry responses by TOC, DIN, TP, log DIN:TP, and log TOC:TP. Atmospheric variables were included as fixed effects, and lake identity was included as a random intercept to account for repeated observations through time within lakes. Except for MAT (approximately normally distributed), variables were log10‐transformed to improve normality (including MAP, Ndep, Sdep, TOC, DIN, and TP; ratios were already analyzed as log10‐transformed DIN:TP and TOC:TP). To account for multiple comparisons, we applied the Holm–Bonferroni correction to the *p*‐values of all MLM evaluating water chemistry variables.

### 2.6 Effects of Water Chemistry Trends on Chlorophyll‐*a* Trends

High temporal‐resolution Chl‐*a* data were available only for the Swedish monitoring lakes (74 lakes) from 1996 onward. To align Chl‐*a* with the harmonized water‐chemistry dataset, which was dominated by autumn data, we used annual autumn data also for Chl‐*a*, but from 1996 to 2019 (24 years). We quantified within‐lake monotonic change in Chl‐*a* with time using Mann–Kendall tests and estimated rates of change (year^−1^) using Sen's slope.

Previous studies have shown that phytoplankton biomass may respond nonlinearly (i.e., unimodally) to browning and associated changes in nutrient availability and stoichiometry (Bergström and Karlsson 2019; Isles et al. 2021). However, our objective was not to estimate a single Chl‐*a*–TOC response function across Fennoscandia. Rather, we evaluated whether lakes experiencing stronger temporal changes in water chemistry and stoichiometry also exhibited stronger monotonic changes in Chl‐*a*. This approach was considered more appropriate because individual subregions encompass only a limited portion of the broader TOC gradient and therefore do not capture the full unimodal Chl‐*a* response observed across northern lakes.

Accordingly, we performed MLM to analyze how Chl‐*a* Sen's slopes were related to Sen's slopes in TOC, DIN, TP, logDIN:TP, and logTOC:TP within each Swedish subregion. Two lakes (Stor‐Arasjön in northwest Sweden and Fagertärn in southeast Sweden) were identified as outliers based on residual histograms and quantile plots and were excluded from the relevant subregional analyses. To account for multiple comparisons, *p*‐values from all Chl‐*a* MLMs were adjusted using the Holm–Bonferroni correction.

### 2.7 Shifts in Nutrient Limitation Regimes Across Fennoscandia

To assess whether lakes shifted between different modes of limitation (P‐, N and P co‐, and N‐limitation), we compared the distributions of lake‐specific 5‐year mean DIN:TP (log‐transformed) and TOC between 1991–1995 and 2015–2019 across Fennoscandia. For Swedish lakes, we also assessed how Chl‐*a* and Chl‐*a*‐trends changed between the first 5 years of the Chl‐*a* record (1996–2000) and the last 5 years (2015–2019). Nutrient limitation categories were defined and inferred using published thresholds: log DIN:TP > 0.9 (P‐limitation), 0.5–0.9 (N and P co‐limitation), and < 0.5 (N‐limitation) (Bergström 2010; Isles et al. 2018).

### 2.8 Statistical Software

Mann–Kendall tests were performed in R (v. 4.2.2; R Core Team 2022) using the trend package. Mixed models were fitted in JMP Pro (v. 15.0.0). Additional analyses were conducted in SigmaPlot (v. 14.0).

## 3 Results

### 3.1 Changes and Trends in Atmospheric Variables Across Fennoscandia

**FIGURE 2:**
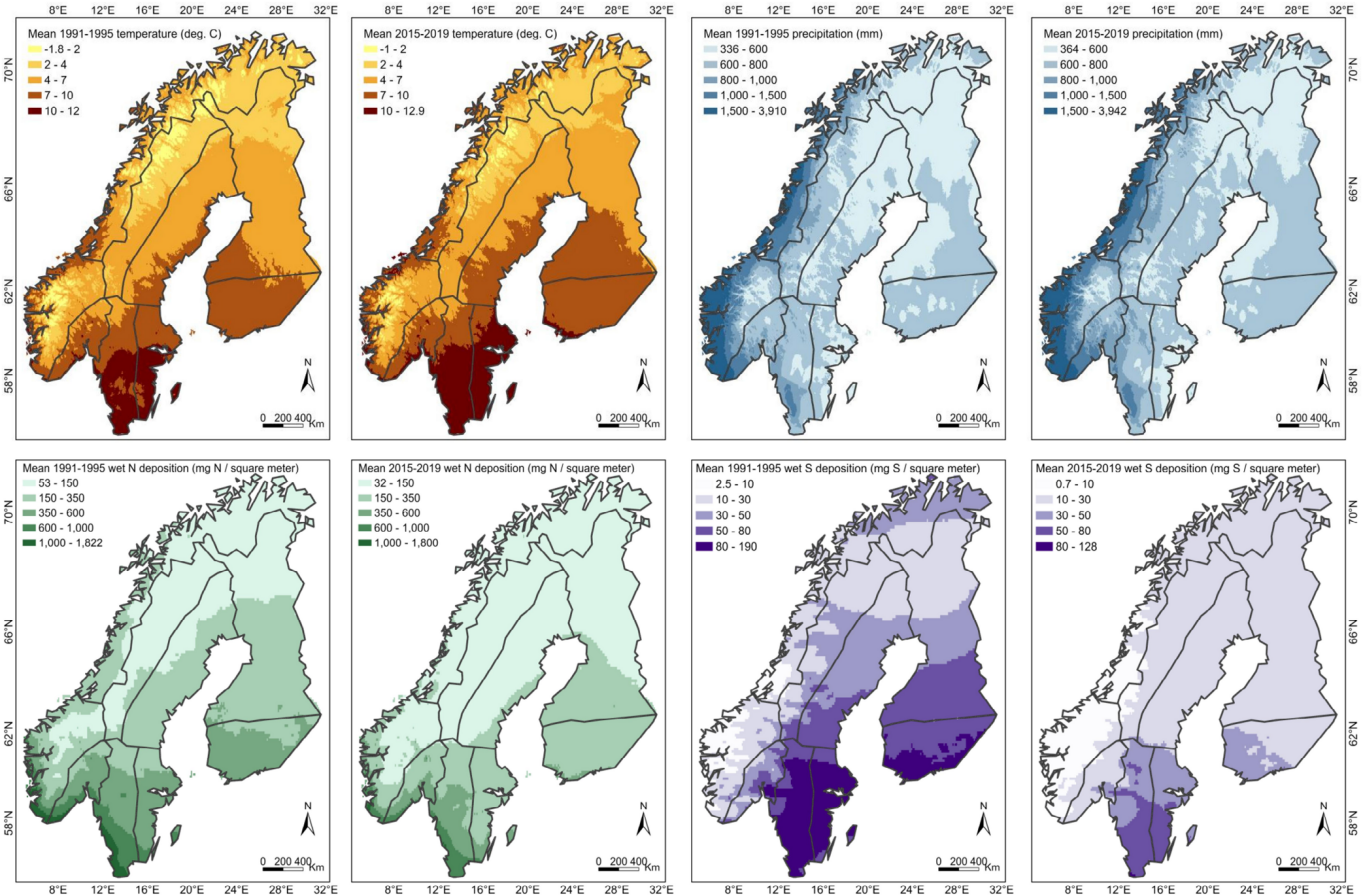
Annual mean maximum air temperature, annual mean precipitation, and annual mean wet N‐ and S‐deposition across Fennoscandia. Borders denote the division of the Fennoscandian subregions (see Figure 1). Data are presented as averages for the first (1991–1995) and last 5 years (2015–2019) of the study period. Note that the minimum and maximum values differ between the left and right panels, illustrating changes for the atmospheric variables between the time periods.

Mean annual maximum temperature (MAT) and mean annual precipitation (MAP) increased across Fennoscandia when comparing 1991–1995 with 2015–2019, with larger increases in the south than in the north (Figure 2; Table 1). Using all years, all subregions showed positive temperature trends, indicating widespread warming (Table 1). Precipitation trends were more heterogeneous, with both negative and positive lake‐level trends (Table 1). Nevertheless, mean MAP increased across Fennoscandia in all subregions except northwestern Sweden, and the strongest increases occurred in southern Norway (Table 1; Figure 2).

**TABLE 1:** Number of lakes with negative and positive Sen's slopes and mean Sen's slopes in atmospheric variables (temperature), precipitation, nitrogen (Ndep), and sulfur deposition (Sdep) for each subregion of Fennoscandia and Fennoscandia as a whole.

Region	Temperature			Precipitation			Ndep			Sdep		
	No. of lakes with negative slopes	No. of lakes with positive slopes	Mean of all slopes (°C year^−1^)	No. of lakes with negative slopes	No. of lakes with positive slopes	Mean of all slopes (mm year^−1^)	No. of lakes with negative slopes	No. of lakes with positive slopes	Mean of all slopes (mg N m^−2^ year^−1^)	No. of lakes with negative slopes	No. of lakes with positive slopes	Mean of all slopes (mg S m^−2^ year^−1^)
Finland north, *n* = 9	0	9	0.052***	1	8	1.025***	9	0	−2.546***	9	0	−5.982***
Finland south, *n* = 10	0	10	0.050***	1	9	1.173***	10	0	−6.804***	10	0	−15.167***
Sweden northeast, *n* = 15	0	15	0.043***	0	15	1.414***	15	0	−3.802***	15	0	−7.834***
Sweden northwest, *n* = 15	0	15	0.041***	5	10	0.209	15	0	−1.919***	15	0	−3.570***
Sweden southeast, *n* = 15	0	15	0.044***	0	15	2.257***	15	0	−6.115***	15	0	−12.764 ***
Sweden southwest, *n* = 30	0	30	0.046***	4	26	2.041***	30	0	−10.204***	30	0	−19.955***
Norway north, *n* = 38	0	38	0.041***	11	27	1.806***	38	0	−4.572***	38	0	−9.967***
Norway southeast, *n* = 13	0	13	0.032***	0	13	7.545***	13	0	−7.441***	13	0	−17.853***
Norway southwest, *n* = 24	0	24	0.029***	0	24	7.70***	24	0	−14.314***	24	0	−25.883***
Fennoscandia, *n* = 169			0.041***			2.911***			−7.033***			−14.194***

*Note:* Only lakes with negative and positive slopes are calculated (i.e., lakes with Sen's slopes = 0.0 are not considered). Mean slope is calculated as average of all Sen's slopes (i.e., negative and positive slopes over all lakes) per subregion for the period 1991 to 2019. Asterisks summarize results of the *t*‐test showing if mean slope is significantly different from 0 (in bold); *p* < 0.01 (***).

Atmospheric nitrogen deposition (Ndep) and sulfur deposition (Sdep) declined markedly between 1991–1995 and 2015–2019, and large parts of Fennoscandia are now characterized by low acidic deposition (Figure 2). The highest present‐day Ndep is concentrated in southern Norway and southern Sweden, whereas the highest present‐day Sdep is concentrated in southern Sweden only (Figure 2). Across lakes and for all subregional means, both Ndep and Sdep exhibited declining trends, with the steepest declines in the southern subregions (Table 1).

### 3.2 Comparison of Lake Water Chemistry Between 1991–1995 and 2015–2019

**FIGURE 3:**
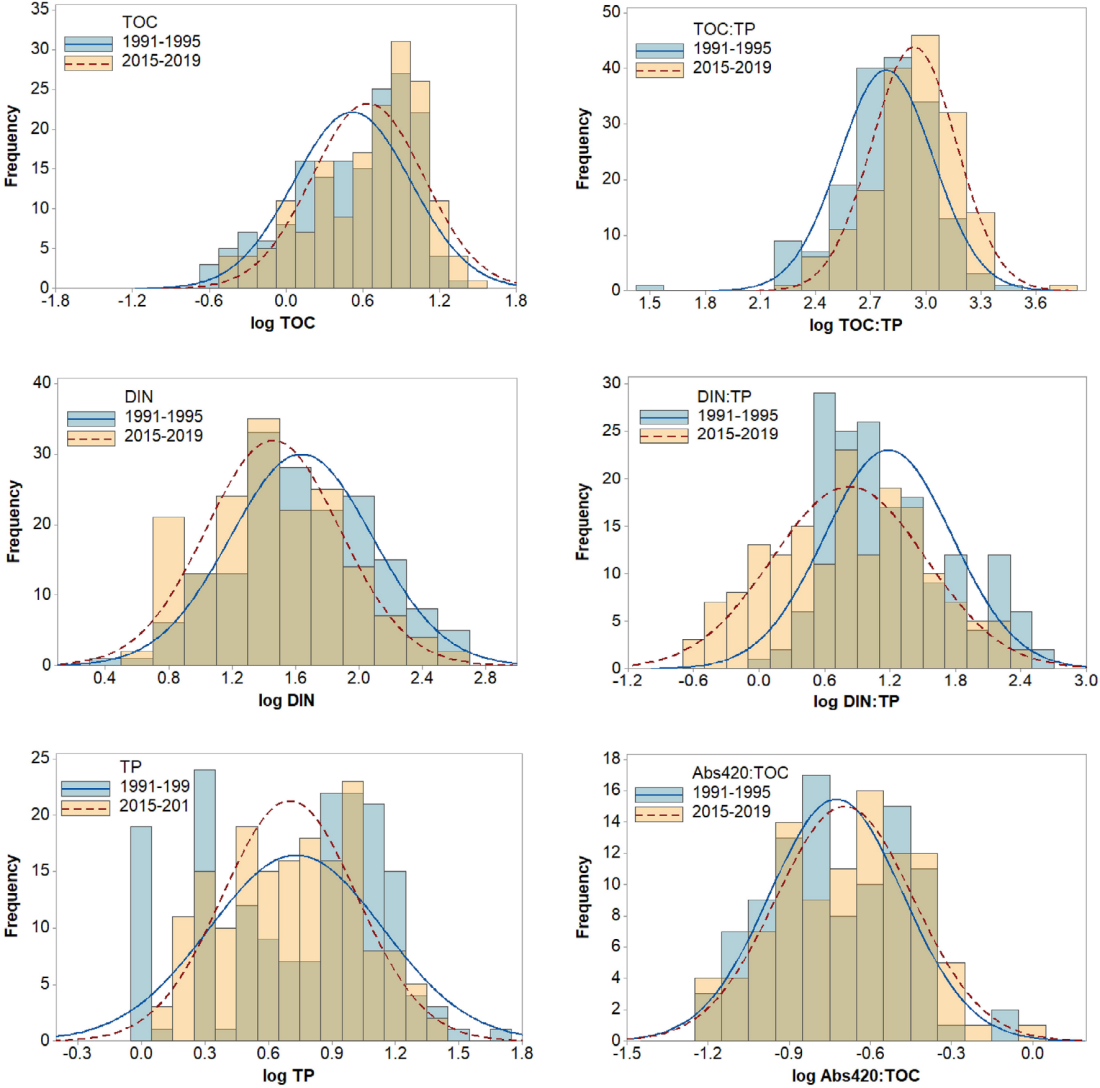
Histograms of the distribution of the means in log TOC (mg L^−1^), log DIN (μg L^−1^), log TP (μg L^−1^), log TOC:TP (mass ratio), log DIN:TP (molar ratio), and log Abs_420_:TOC in Fennoscandian lakes in the first (1991–1995 in blue) and last 5 years (2015–2019 in light orange) of the study period. Bins with overlapping data for the two periods are shown as brown.

Lake water chemistry changed significantly across Fennoscandia between 1991–1995 and 2015–2019 (Figure 3). TOC increased (*p* < 0.001; medians 4.2 mg L^−1^ vs. 5.9 mg L^−1^) and DIN declined (*p* < 0.001; medians 44 μg L^−1^ vs. 29 μg L^−1^). In contrast, TP did not change significantly between time periods (*p* = 0.19; medians 7.2 μg L^−1^ vs. 4.9 μg L^−1^). Although median TP did not shift significantly, the TP distribution narrowed: fewer lakes had very low TP (< 2.5 μg L^−1^; log TP < 0.4) or high TP (> 10 μg L^−1^; log TP > 1) in 2015–2019 than in 1991–1995 (Figure 3).

Stoichiometric ratios shifted consistently with these concentration changes between the two time periods. The log TOC:TP increased (2.81 to 2.97, *p* < 0.001) and log DIN:TP declined (1.04 to 0.81, *p* < 0.001), whereas log Abs_420_:TOC (Sweden only) did not change significantly (0.17 to 0.22, *p* = 0.37) (Figure 3).

**FIGURE 4:**
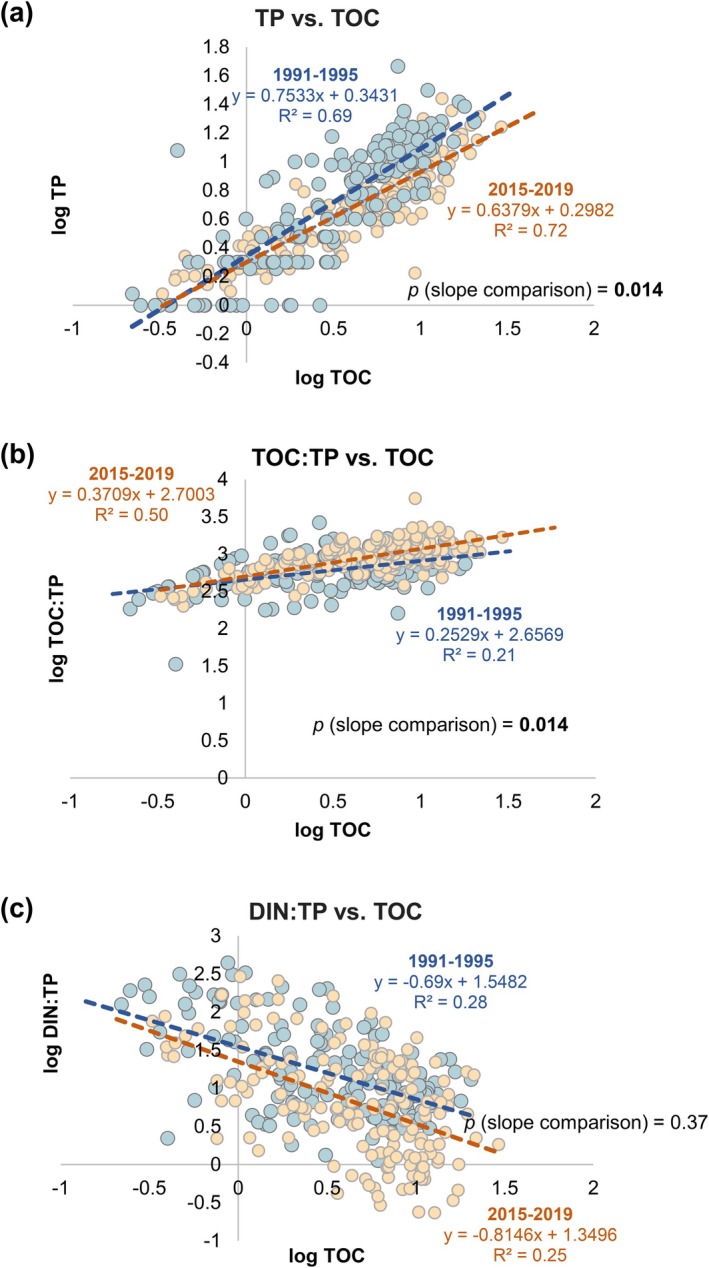
(a) log TP (μg L^−1^), (b) log TOC:TP (mass ratio), and (c) log DIN:TP (molar ratio) plotted against log TOC (mg L^−1^) for Fennoscandian lakes showing relative changes in water chemistry between the first (1991–1995 in blue) and last 5 years (2015–2019 in light orange) of the study period. The water chemistry parameters are plotted as mean values for 1991–1995 and 2015–2019 periods accordingly. *p* (slope comparison) shows if the slopes of two regression lines are significantly different (in bold) from each other with the significance level set at *p* < 0.05. The *p* is calculated with the analysis of covariance (ANCOVA).

TOC and TP were strongly positively correlated in both periods; however, the relationship differed significantly between periods (interaction *p* = 0.014), with a steeper TOC–TP slope in 1991–1995 than in 2015–2019 (Figure 4a), indicating that the spatial TOC–TP pattern changed over time. Similarly, log TOC:TP increased with TOC in both periods, but the slope differed between periods (*p* = 0.014), with higher log TOC:TP per unit TOC in 2015–2019 (Figure 4b). The log DIN:TP declined with increasing TOC in both periods, but the slopes did not differ (*p* = 0.367) (Figure 4c), indicating that the TOC–DIN:TP spatial relationship was stable, even though lakes shifted toward higher TOC and lower DIN:TP in the most recent period 2015–2019.

### 3.3 Trends in Water Chemistry in Fennoscandian Subregions

**FIGURE 5:**
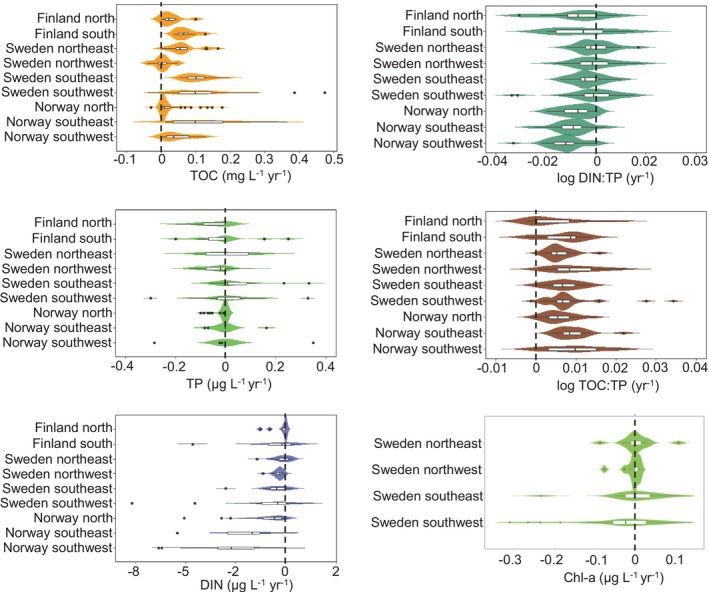
Violin plots showing distributions of changes in water chemistry parameters per year that are, in TOC (mg L^−1^ year^−1^), TP (μg L^−1^ year^−1^), DIN (μg L^−1^ year^−1^), log DIN:TP (molar ratio), and log TOC:TP (mass ratio), and in phytoplankton biomass expressed as chlorophyll‐*a* (Chl‐*a*; μg L^−1^ year^−1^) in each of the Fennoscandian subregions. Changes in water chemistry parameters are calculated as Sen's slopes for individual lakes over the entire study period (1991–2019) and for Chl‐*a* over the 1996–2019 period.

Long‐term trends, including all 29 years, in water chemistry varied among subregions (Figure 5; Table 2). Per region, TOC increased in all subregions except northwestern Sweden and in total 153 lakes showed positive trends with only 7 lakes showing negative trends. The strongest TOC increases occurred in southeastern and southwestern Sweden and southeastern Norway, exceeding 0.1 mg L^−1^ year^−1^ (≈3 mg L^−1^ over 29 years).

**TABLE 2:** Number of lakes with negative and positive Sen's slopes and mean Sen's slopes of TOC, TP, DIN, log DIN:TP and log TOC:TP per region.

Region	TOC			TP			DIN			Log DIN:TP			Log TOC:TP		
	No. of lakes with negative slopes	No. of lakes with positive slopes	Mean of all slopes (mg L^−1^ year^−1^)	No. of lakes with negative slopes	No. of lakes with positive slopes	Mean of all slopes (μg L^−1^ year^−1^)	No. of lakes with negative slopes	No. of lakes with positive slopes	Mean of all slopes (μg L^−1^ year^−1^)	No. of lakes with negative slopes	No. of lakes with positive slopes	Mean of all slopes (year^−1^)	No. of lakes with negative slopes	No. of lakes with positive slopes	Mean of all slopes (year^−1^)
Finland north, *n* = 9	0	7	0.029**	4	0	−0.053*	3	0	−0.253	6	1	−0.008*	1	5	0.004
Finland south, *n* = 10	0	10	0.071***	3	2	0.001	6	3	−0.678	7	3	−0.005	1	9	0.007***
Sweden northeast, *n* = 15	0	14	0.067***	5	5	0.01	9	4	−0.206	8	7	0.0003	0	15	0.006***
Sweden northwest, *n* = 15	5	8	0.005	10	1	−0.037**	14	0	−0.377***	9	6	−0.0002	0	15	0.013***
Sweden southeast, *n* = 15	0	15	0.105***	4	8	0.049*	10	2	−0.552**	10	3	−0.003*	0	14	0.007***
Sweden southwest, *n* = 30	1	29	0.123***	10	14	0.007	21	7	−0.788**	17	13	−0.001	1	29	0.008***
Norway north, *n* = 38	1	33	0.028***	7	0	−0.011**	36	1	−0.884***	32	0	−0.008***	1	35	0.006***
Norway southeast, *n* = 13	0	13	0.133***	2	1	0.001	12	1	−1.952***	12	1	−0.01***	0	13	0.01***
Norway southwest, *n* = 24	0	24	0.050***	3	1	0.001	23	1	−3.441***	22	2	−0.012***	1	22	0.01***
Fennoscandia, *n* = 169			0.068***			−0.004			−1.015***			**0.000**			0.008***

*Note:* Only lakes with negative and positive slopes are calculated (i.e., lakes with Sen's slopes = 0.0 are not considered). Mean slope is calculated as average of all Sen's slopes (i.e., negative and positive slopes over all lakes) per subregion for the period 1991 to 2019. Asterisks summarize results of the *t*‐test showing if mean slope is significantly different from 0 (in bold); *p* < 0.01 (***), *p* < 0.05 (**), and *p* < 0.1 (*).

TP trends were more variable across subregions (Table 2; Figure 5), with 48 lakes showing negative trends and 32 lakes showing positive trends. Consistent TP declines occurred in northern Fennoscandia, with mean slopes from −0.011 to −0.053 μg L^−1^ year^−1^ (−0.3 to −1.5 μg L^−1^ over 29 years). In contrast, southeastern Sweden showed significantly increasing TP (0.049 μg L^−1^ year^−1^), coinciding with increasing TOC (Table 2). DIN generally declined across subregions, with particularly strong declines in southern Sweden and Norway (slopes −0.552 to −3.441 μg L^−1^ year^−1^, −16 to −100 μg L^−1^ over 29 years) (Table 2; Figure 5). Across Fennoscandia as a whole, TOC increased significantly and DIN declined significantly (Table 2).

Stoichiometric trends were strongly directional. The log DIN:TP declined in most lakes (123 negative vs. 35 positive trends), with the steepest declines in Norway (means −0.008 to −0.012 year^−1^, −0.2 to −0.35 over 29 years). Declines were also evident in northern Finland (−0.008 year^−1^, ≈ −0.2) and southeastern Sweden (−0.003 year^−1^, ≈ −0.09). The log TOC:TP increased in all subregions (means 0.006–0.013 year^−1^, 0.174–0.377 over 29 years), with the largest mean increase in northwestern Sweden (Table 2; Figure 5). Across Fennoscandia overall, log TOC:TP increased significantly (Table 2).

**FIGURE 6:**
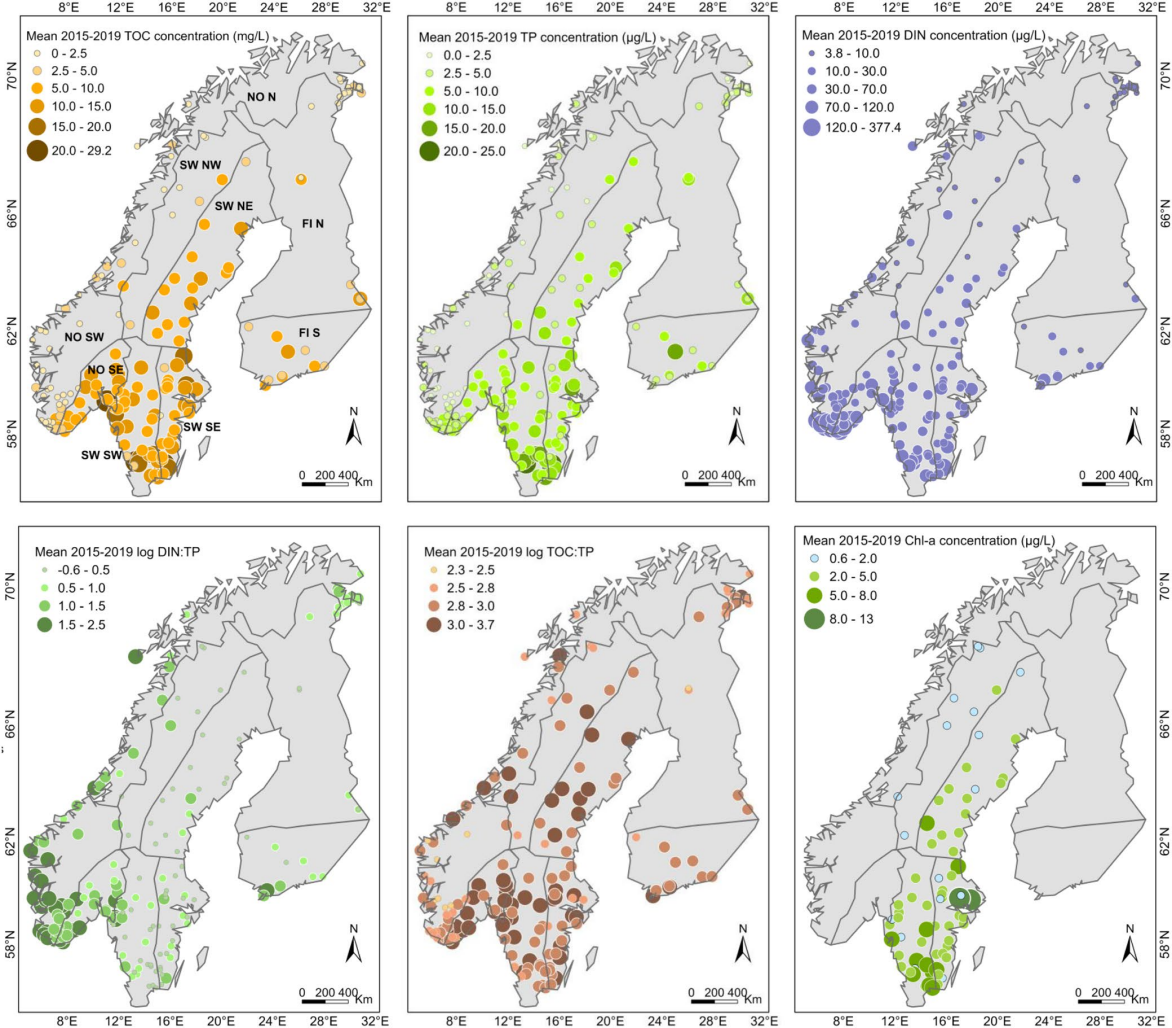
Spatial distribution of mean lake water chemistry—TOC, TP, DIN, log DIN:TP (molar ratio), and log TOC:TP (mass ratio)—and chlorophyll‐*a* (Chl‐*a*) in Fennoscandian and Swedish (for Chl‐*a*) subregions, based on average values from the last 5 years (2015–2019) of the study period.

Despite these temporal trends, substantial spatial heterogeneity remained (Figure 6). TOC and TP generally increased from Norway toward northeastern Fennoscandia (toward Finland). DIN was higher in southern Fennoscandia, with highest concentrations in southwestern Norway. The log TOC:TP was lowest in southwestern Norway and increased northeastward, whereas log DIN:TP was highest in Norway and decreased eastward toward Sweden and Finland (Figure 6).

### 3.4 Spatiotemporal Effects of Atmospheric Variables on Lake Water Chemistry

**FIGURE 7:**
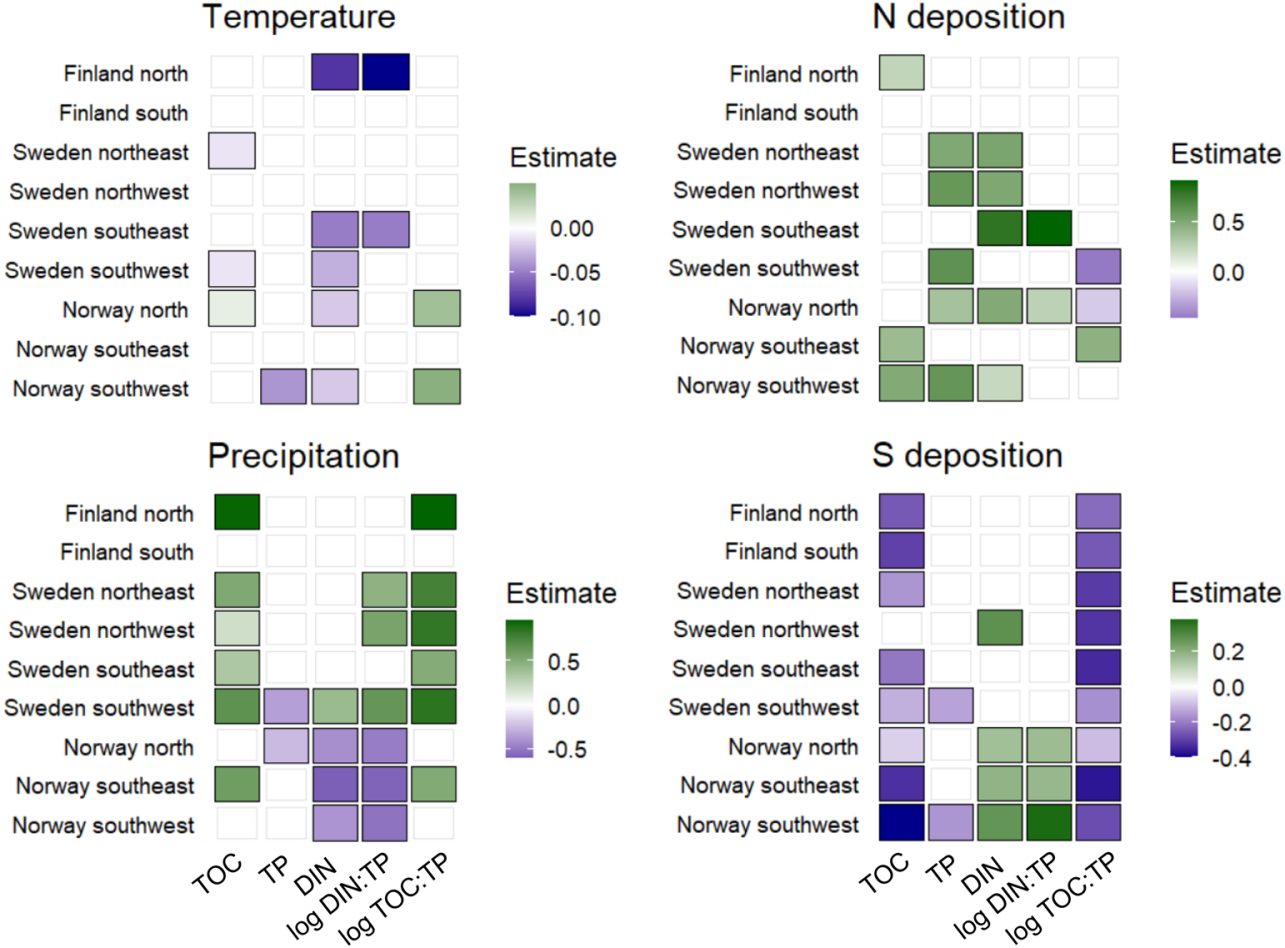
Heatmap showing the results of mixed linear models for the 1991–2019 time series of lake water chemistry and atmospheric variables (temperature, precipitation, N deposition and S deposition) for each subregion of Fennoscandia. Colors represent positive and negative effects of atmospheric variables on water chemistry, which are based on coefficient estimates (see Table S1 for details). Green = positive effect, blue = negative effect; white = non‐significant effect (*p* ≥ 0.05). The color gradient represents the strength of the estimates (the closer to 0 the weaker the effect).

The MLMs revealed multiple significant associations between atmospheric variables and lake water chemistry within subregions (Figure 7; Table S1). In many models, the random lake effect was significant, indicating heterogeneous lake‐specific responses to atmospheric forcing within subregions (Tables S1 and S2).

Across most subregions, increasing TOC (browning) was associated with declining sulfur deposition (negative Sdep effects) and increasing precipitation (positive precipitation effects) (Figure 7). Ndep was positively associated with TOC in southern Norway and northern Finland. For TP, Ndep showed positive effects in five of nine subregions, indicating lower lake TP under lower Ndep. In northern Norway and southwestern Sweden, TP was also negatively related to precipitation. Patterns in log TOC:TP largely mirrored those for TOC, with log TOC:TP generally negatively related to Sdep and positively related to precipitation (Figure 7; Tables S1 and S2).

DIN showed more variable atmospheric associations among subregions. As expected, DIN was positively related to Ndep in many subregions (lower DIN under lower Ndep). In southern Sweden, northern Finland, and northern and southwestern Norway, DIN was also negatively related to temperature (lower DIN under warming). In Norway, DIN was negatively related to precipitation, whereas in southwestern Sweden DIN was positively related to precipitation (Figure 7; Tables S1 and S2). Because DIN was more consistently associated with atmospheric variables than TP, log DIN:TP often showed similar atmospheric associations to DIN.

### 3.5 Effects of Water Chemistry on Chlorophyll‐*a* Trends (Sweden)

Across Swedish subregions (1996–2019), lakes exhibited both increasing and declining trends in Chl‐*a* (Figure 5). The strongest changes occurred in southern Sweden. In southwestern Sweden (*n* = 29 lakes), 19 lakes showed declining trends and 10 lakes showed increasing trends; in southeastern Sweden (*n* = 15 lakes), 5 lakes showed declining trends and 7 lakes showed increasing trends. In northern Sweden, Chl‐*a* trends were generally weaker but remained mixed (northeast Sweden, *n* = 15 lakes: 5 negative and 6 positive; northwestern Sweden, *n* = 15 lakes: 4 negative and 5 positive).

Comparisons between the periods 1996–2000 and 2015–2019 revealed only minor changes in mean Chl‐*a* across most subregions. Mean Chl‐*a* increased slightly in northwestern Sweden (from 1.8 to 1.9 μg L^−1^) and northeastern Sweden (from 3.1 to 3.3 μg L^−1^), while remaining essentially unchanged in southeastern Sweden (4.2 μg L^−1^). The exception was southwestern Sweden, where mean Chl‐*a* declined from 4.6 to 3.9 μg L^−1^ and was significantly lower during 2015–2019 than during 1996–2000 (paired *t*‐test, *p* = 0.041).

The MLM results indicated that the lake TP slopes were positively associated with the Chl‐*a* slopes in southeastern Sweden (Table 3). In southwestern Sweden, the lake TOC slopes were negatively correlated with the Chl‐*a* slopes, while the log TOC:TP slopes showed a positive association with the Chl‐*a* slopes (Table 3). In this subregion, the Chl‐*a* slopes were also positively associated with the DIN slopes and negatively correlated with the log DIN:TP slopes at *p* < 0.1 (*p* = 0.066–0.070). In northeastern and northwestern Sweden, the TP slopes showed weaker associations with the Chl‐*a* slopes (*p* < 0.1; *p* = 0.054–0.085), and in northwestern Sweden, the log DIN:TP slopes tended to have a positive association with the Chl‐*a* slopes (*p* = 0.09).

**TABLE 3:** Results of mixed linear models for the trends (i.e., Sen's slopes) of lake water chemistry and chlorophyll a (Chl‐*a*) concentrations for each Swedish subregion.

Subregion	Fixed	Chl‐*a*_slope_	*p*
		Estimate	
Sweden northeast	Model		0.453
	Intercept	0.031	0.483
	TOC_slope_	−0.599	0.267
	TP_slope_	**0.443**	0.085*
	DIN_slope_	0.024	0.526
	Log DIN:TP_slope_	−4.300	0.207
	Log TOC:TP_slope_	2.618	0.537
Sweden northwest	Model		0.214
	Intercept	−0.007	0.346
	TOC_slope_	0.260	0.160
	TP_slope_	**−0.110**	0.054*
	DIN_slope_	−0.017	0.326
	Log DIN:TP_slope_	**1.157**	0.090*
	Log TOC:TP_slope_	−0.272	0.445
Sweden southeast	Model		0.129
	Intercept	0.005	0.909
	TOC_slope_	0.212	0.451
	TP_slope_	**0.254**	0.035**
	DIN_slope_	0.012	0.593
	Log DIN:TP_slope_	1.482	0.597
	Log TOC:TP_slope_	−1.544	0.671
Sweden southwest	Model		0.060*
	Intercept	−0.032	0.370
	TOC_slope_	**−0.404**	0.035**
	TP_slope_	0.188	0.144
	DIN_slope_	**0.047**	0.066*
	Log DIN:TP_slope_	**−8.683**	0.070*
	Log TOC:TP_slope_	**8.535**	0.043**

*Note:* Negative (or positive) coefficient estimates of water chemistry trends indicate negative (or positive) effects on the Chl‐*a* trends. Note that one lake each in Sweden northwest (Stor‐Arasjön) and southeast (Fagertärn) were identified as outliers and excluded from the analysis. *p* values are marked in bold with *p* < 0.05 (**), and *p* < 0.1 (*), respectively.

### 3.6 Changes in Nutrient Limitation Regimes Across Fennoscandia

**FIGURE 8:**
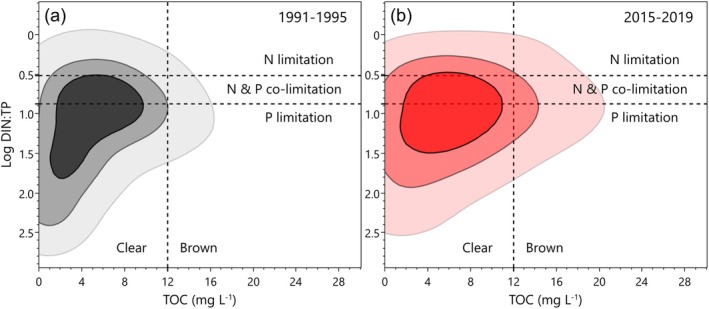
Lake density plots based on water DIN:TP molar ratio and TOC concentration of all study lakes in (a) 1991–1995 and (b) 2015–2019. Contours are bivariate nonparametric density surface fits indicating the density of data points: (a) 100% (light grey), 67% (grey), and 33% (dark grey) density of the lakes; (b) 100% (light red), 67% (red), and 33% (dark red) density of the lakes. The vertical broken line indicates the TOC threshold above which lakes shift from clear‐water, nutrient‐limited conditions to brown, light‐limited conditions (Solomon et al. 2015). Horizontal broken lines indicate log DIN:TP thresholds of nutrient limitation regimes for phytoplankton: > 0.9 for P‐limitation, 0.5–0.9 for N and P co‐limitation, and < 0.5 for N‐limitation (Bergström 2010; Isles et al. 2018).

We evaluated shifts in inferred phytoplankton nutrient limitation by tracking changes in TOC and log DIN:TP between two 5‐year periods (1991–1995 and 2015–2019) across Fennoscandia and by subregion (Figures 8 and S1; Tables 2 and 4). Overall, lakes shifted toward higher TOC and toward more potential N‐limited or N and P co‐limited conditions (Figure 8).

**TABLE 4:** Changes in TOC (mg L^−1^), log DIN:TP (molar ratios) and phytoplankton nutrient limitation regime between the time periods 1991–1995 and 2015–2019 (means with standard deviation within parenthesis) across the Fennoscandian subregions, as well as changes in phytoplankton biomass (expressed as chlorophyll‐*a* [Chl‐*a*; μg L^−1^]) and the mean Chl‐*a* Sen slopes across the Swedish subregions between the time periods of 1996–2000 and 2015–2019 (means with standard deviation within parenthesis).

Region	Log DIN:TP	Log DIN:TP	TOC	TOC	Nutrient limitation change	DIN‐trends	TP‐trends	TOC‐trends	Chl‐*a* trends	Chl‐*a*	Chl‐*a*	Chl‐*a* (year^−1^)
	1991–1995	2015–2019	1991–1995	2015–2019						1996–2000	2015–2019	
FI‐N	0.6 (0.3)	0.5 (0.3)	3.6 (3.5)	4.1 (3.3)	NP → N	—	—*	+**				
FI‐S	1.1 (0.5)	0.9 (0.5)	4.2 (2.4)	5.6 (3.0)	P → NP	—	+	+***				
SWE‐NE	0.7 (0.3)	0.8 (0.3)	8.4 (2.4)	9.9 (3.1)	NP	—	+	+***	+	3.1 (1.9)	3.3 (1.8)	0.005
SWE‐NW	0.6 (0.3)	0.7 (0.3)	4.9 (3.7)	4.7 (2.7)	NP	—***	—**	+	—	1.8 (1.0)	1.9 (1.7)	−0.004
SWE_SE	0.9 (0.4)	0.9 (0.4)	7.7 (3.1)	9.7 (2.9)	P and NP	—**	+*	+***	No	4.2 (3.0)	4.2 (3.3)	0.000
SWE_SW	1.0 (0.4)	1.0 (0.4)	9.0 (5.0)	11.3 (6.5)	P towards NP	—**	+	+***	—	4.6 (3.5)	3.9 (2.6)	−0.035
NO‐N	1.4 (0.5)	1.2 (0.5)	2.7 (3.0)	3.4 (4.0)	P towards NP	—***	—**	+***				
NO‐SE	1.6 (0.4)	1.4 (0.4)	5.3 (3.7)	8.4 (5.2)	P towards NP	—***	+	+***				
NO‐SW	2.1 (0.4)	1.7 (0.5)	1.8 (1.7)	3.0 (2.4)	P towards NP	—***	+	+***				
Fennoscandia	1.2 (0.6)	1.1 (0.5)	5.2 (4.3)	6.6 (5.3)	P towards NP	—***	—	+***				

*Note:* The DIN‐, TP‐, TOC‐ trends denote the direction of trends of these parameters based on their Sens slopes in Table 2. Thresholds in log DIN:TP for phytoplankton N‐, NP‐ and P‐limitation are from Bergström (2010) and Isles et al. (2018); that is, for N‐limitation the log DIN:TP < 0.5, for NP‐limitation the log DIN:TP 0.5–0.9, for P‐limitation the log DIN:TP > 0.9. Asterisks summarize results of the *t*‐test showing if mean slope is significantly different from 0 (in bold); *p* < 0.01 (***), *p* < 0.05 (**), and *p* < 0.1 (*) (see Table 2).

Using the 12 mg L^−1^ TOC threshold to select clear from brown lakes, the number of lakes below versus above this threshold changed from 155 and 14 (1991–1995) to 146 and 23 (2015–2019). Mean TOC values within these groups were 4 and 14 mg L^−1^ in 1991–1995 and 5 and 15 mg L^−1^ in 2015–2019. Consistent with declining log DIN:TP, the number of lakes classified and inferred as N‐limited (log DIN:TP < 0.5) increased from 7 to 12, N and P co‐limited (log DIN:TP 0.5–0.9) increased from 49 to 59, and P‐limited (log DIN:TP > 0.9) declined from 113 to 98 between 1991–1995 and 2015–2019 (Figure 8), indicating a net shift toward more potential N‐ and N and P co‐limitation.

However, trends were not uniform among subregions (Figures 5, 8, and S1; Tables 2 and 4). Apart from northwestern Sweden, subregional means indicate browning across regions, with the strongest TOC increases in southern Sweden and southeastern Norway (Table 4; Figure S1). Over the same period, mean log DIN:TP declined in Finland and Norway, remained approximately unchanged in southern Sweden, and increased in northern Sweden. These subregional log DIN:TP shifts imply transitions in inferred limitation regime in some cases: northern Finland shifted from inferred N and P co‐limitation toward potential N limitation (0.6 to 0.5), and southern Finland shifted from inferred P limitation toward potential N and P co‐limitation (1.1 to 0.9). Northern Sweden remained N‐limited to N and P co‐limited despite a slight increase in log DIN:TP (inferred boundary 0.6–0.8), and southeastern Sweden remained near the potential P‐limited or the N and P co‐limited inferred boundary (~0.9). In contrast, southwestern Sweden and Norway remained potential P‐limited (inferred boundary > 0.9), although Norwegian lakes had lower log DIN:TP in 2015–2019 than in 1991–1995 (Table 4). Changes in mean Chl‐*a* and Chl‐*a*‐trends (Sen slopes) for Swedish subregions (1996–2000 vs. 2015–2019) are summarized in Table 4.

## 4 Discussion

### 4.1 Atmospheric Controls on Browning and Nutrient Trajectories

Most Fennoscandian lakes are becoming browner and increasingly nutrient‐depleted, with rising TOC and declining DIN and, in some regions, declining TP. These shifts have increased TOC:TP and generally lowered DIN:TP, linking lake chemical change to broad‐scale atmospheric drivers (temperature, precipitation, and declining N and S deposition). Overall, lakes are moving toward more potential N and P co‐limited or N‐limited conditions as browning intensifies and DIN:TP declines, with regionally distinctive responses in phytoplankton biomass.

Browning (TOC) trends were consistent across Fennoscandia, except for northwestern Sweden, and were primarily associated with declining S deposition. The strongest TOC increases occurred in historically acidified areas (all subregions except northwestern Sweden and northern Finland), supporting continued recovery from acidification as sulfur deposition declines (Monteith et al. 2007; Erlandsson et al. 2008; Räike et al. 2024). Increasing precipitation also coincided with TOC increases across many subregions, consistent with enhanced terrestrial organic matter export to lakes under wetter conditions (Freeman et al. 2001; Erlandsson et al. 2008; de Wit et al. 2016; Imtiazy et al. 2025). In contrast, TOC showed weak associations with temperature and N deposition, implying a lesser role for warming‐driven terrestrial C fixation and land‐use change in TOC dynamics over the 29‐year period assessed (Finstad et al. 2016; Räike et al. 2024).

TP trends were more variable across subregions. Declining TP dominated in northern Fennoscandia, whereas in southern subregions browning appeared to mitigate TP declines in support of previous studies (Kortelainen et al. 2006; Huser et al. 2018; Isles et al. 2018; Nilsson et al. 2024). TP trends were mainly associated with N deposition, consistent with increased watershed P retention during acidification recovery (Huser et al. 2018). Other atmospheric variables were weakly related to TP, and in Finland TP trends were not associated with any atmospheric variables. Warming‐induced catchment greening can also enhance terrestrial P retention, especially in mountain landscapes, reducing P inputs to clear‐water, low‐DOC lakes (Goedkoop et al. 2025). In line with this, declining TP occurred primarily in subregions with low‐TOC lakes (northern Finland, northwestern Sweden, and northern Norway), suggesting vegetation development as an important driver (Goedkoop et al. 2025). The drivers of long‐term declines in TP are likely region‐ and catchment‐specific (Stetler et al. 2021; Isles et al. 2023). Despite variable TP change, TOC:TP increased consistently across Fennoscandia and was linked primarily to S deposition and secondarily to precipitation, supporting the rising carbon‐per‐phosphorus and temporal decoupling between C and P also reported for U.S. lakes (Stetler et al. 2021).

DIN declines were pervasive and strongest in southern Norway. These trends tracked declining N deposition, consistent with reduced direct deposition to lake surfaces (Isles et al. 2018) and/or reduced catchment DIN delivery (Bergström et al. 2008; Hessen 2013). In Norway, DIN also declined with increasing precipitation, consistent with dilution (Hessen et al. 2009). Temperature was negatively related to DIN, particularly in northern Finland, consistent with enhanced terrestrial N retention under catchment greening and increased forest biomass (Hessen et al. 2009; Lucas et al. 2016; Goedkoop et al. 2025). Reduced DIN may also reflect enhanced denitrification under higher TOC (Weyhenmeyer and Jeppesen 2009). Continued warming, increasing precipitation, and declining N deposition therefore suggest continued downward pressure on lake DIN.

Because TP trends were variable, DIN:TP trends were less uniform than DIN. Still, declining DIN:TP clearly dominated (123 negative vs. 35 positive trends), producing significant declines in Norway, southeastern Sweden, and northern Finland. Declining DIN:TP was primarily related to declining atmospheric acid deposition and increasing precipitation.

### 4.2 From Chemistry to Limitation: Shifts Are Expected but Not Uniform

The combined increase in TOC and decline in DIN, together with generally declining DIN:TP, indicate ongoing shifts in phytoplankton nutrient limitation regimes toward more potential N and P co‐limited and N‐limited conditions across Fennoscandia. The magnitude and direction of ecological responses should vary because phytoplankton biomass depends on both a lake's position along the TOC gradient and its nutrient limitation regime, reflected by DIN:TP (Bergström et al. 2022). Under this framework, browning can stimulate biomass in relatively clear lakes via increased nutrient supply but suppress biomass at higher TOC where light limitation dominates, and these outcomes should differ among regions due to spatial variability in TOC and DIN:TP and in their long‐term trajectories (Kelly et al. 2018; Bergström and Karlsson 2019; Isles et al. 2021), potentially resulting in region‐specific impacts on carbon and nutrient transfer in pelagic food chains (Bergström et al. 2026).

Although TOC:TP increased between 1991–1995 and 2015–2019, TOC and TP remained spatially correlated, indicating that browning still tends to increase TP, but at a lower rate in 2015–2019 than in 1991–1995. Abs_420_:TOC did not change between periods in Swedish lakes, implying that long‐term light changes reflect TOC concentration change rather than altered organic matter optical quality.

### 4.3 Regional Phytoplankton Responses in Sweden and Implications for Fennoscandia

Phytoplankton biomass (Chl‐*a*) trends were mixed across Sweden and differed in their associations with water‐chemistry trends, including TOC and DIN:TP. The strongest positive and negative trends occurred in southern Sweden, where browning was consistent and DIN declined, but DIN:TP changed little due to browning partially mitigating TP declines. These lakes therefore remained primarily potential P‐limited in the southwest (DIN:TP = 1.0) and moved toward potential N and P co‐limitation in the southeast (DIN:TP = 0.9), with sufficient DIN for phytoplankton growth under further browning and TP inputs.

In southwestern Sweden, Chl‐*a* trends were primarily negatively associated with TOC and secondarily (and more weakly) negatively associated with DIN:TP. This pattern is consistent with the unimodal framework in which browning and associated TP inputs can promote biomass increases below the TOC threshold, but biomass declines above 10–12 mg L^−1^ as light limitation dominates. Southwestern Sweden was also the only subregion with significantly lower mean Chl‐*a* in 2015–2019 than in 1996–2000 and with a mean negative Chl‐*a* trend, consistent with browning pushing many lakes onto the dim, high‐TOC‐brown side of the response curve. The negative Chl‐*a*–DIN:TP association is also consistent with primarily potential P‐limited conditions.

In southeastern Sweden, Chl‐*a* trends were primarily positively associated with TP trends. Lakes here were less brown and experienced weaker browning than in the southwest, leaving more systems in clear‐water conditions where increasing TP can stimulate biomass under relatively high DIN:TP. Mean Chl‐*a* did not change between 1996–2000 and 2015–2019, and mean trends were near zero, consistent with mixed positive and negative lake‐level trends.

In northern Sweden, lakes are more often N‐limited or N and P co‐limited and have lower DIN:TP overall (Isles et al. 2020; Bergström et al. 2008, 2022). Browning dominated in the northeast (higher TOC), whereas in the northwest browning was weaker and more inconsistent, and DIN and TP declines occurred in lower‐TOC lakes (see also Johnson et al. 2025). Chl‐*a* trends were weaker than in southern Sweden, as expected under lower DIN:TP. In northwestern Sweden, Chl‐*a* was primarily negatively associated with TP trends and secondarily positively associated with DIN:TP trends, consistent with expected responses in phytoplankton during N‐limited conditions (Bergström et al. 2022). Mean Chl‐*a* trends were slightly negative. In northeastern Sweden, Chl‐*a* trends were positively associated with TP, consistent with some biomass stimulation with browning, but likely weaker than in the south due to lower DIN availability; mean trends were slightly positive. Both northern subregions of Sweden showed slight but non‐significant increases in mean Chl‐*a* between 1996–2000 and 2015–2019.

Finally, Swedish lakes span ultraoligotrophic clear‐water mountain lakes to dystrophic brown‐water forest lakes, overlapping gradients also present in Norway and Finland. Thus, Swedish subregional patterns provide a reasonable basis for expectations across Fennoscandia. In Norway, increasing phytoplankton biomass may dominate with further browning because lakes remain largely potential P‐limited (DIN:TP > 0.9) and have not yet become extremely brown or dark (< 12 mg L^−1^). Browning has been most prominent in southeastern Norway, where phytoplankton biomass is therefore likely to have increased most with associated TP inputs. In Finland, phytoplankton biomass is also likely to increase with browning, especially in southern Finland where DIN:TP is higher.

### 4.4 Limitations

Despite covering an extensive timespan for a high number of lakes, this study also comes with some limitations. First, phytoplankton biomass analyses were restricted to Swedish lakes with long‐term Chl‐*a* data, so implications for Norway and Finland should be treated as informed expectations rather than direct evidence. Second, Chl‐*a* trends were derived from autumn samples, which may not capture summer maxima and may differ from summer‐based trends, contributing to differences in trend strength among studies. Nevertheless, our autumn Chl‐*a* trends are broadly consistent with those reported by Paltsev et al. (2024) using summer mean Chl‐*a* concentrations (June–September) and by Johnson et al. (2025) using phytoplankton cell counts collected in August. Both studies similarly indicate contrasting phytoplankton trajectories between southern and northern Sweden, with southern regions exhibiting both increasing and decreasing trends among lakes and northern regions showing predominantly increasing trends. This spatial variability has been linked to regional differences in phytoplankton community composition and environmental conditions (Paltsev et al. 2024; Johnson et al. 2025). Finally, the atmospheric variables represent broad‐scale forcing but not catchment properties (e.g., vegetation change, soils, hydrological pathways) that likely modulate regional responses, particularly for TP. Continued long‐term monitoring that couples deposition and climate forcing with catchment attributes and seasonally resolved biological metrics will be essential for resolving mechanisms behind phosphorus change and for improving predictions under continued browning and nutrient rebalancing.

## 5 Conclusion

Long‐term monitoring across Fennoscandia revealed a coherent shift toward browner and more N‐depleted lake states, with rising TOC, declining DIN, and regionally mixed TP change. These trajectories have increased TOC:TP and generally lowered DIN:TP, consistent with a movement toward more frequent N and P co‐limitation and N‐limitation as browning intensifies. The patterns point to combined roles of acidification recovery and increasing precipitation in promoting browning, and declining nitrogen deposition (and in some regions warming) in reducing lake DIN, while long‐term TP change remains more region‐ and catchment‐specific. Ecologically, continued TOC increases are likely to push more lakes toward stronger light limitation, while concurrent DIN declines reduce the potential for browning‐linked TP inputs to translate into higher phytoplankton biomass, especially where lakes shift onto the high‐TOC, low‐light side of the unimodal biomass response. Beyond biomass, shifting stoichiometry may also alter phytoplankton biochemical (e.g., fatty acids) (Lau et al. 2021) and mineral quality (Bergström and Karlsson 2019) and thus energy transfer to zooplankton (Elser et al. 2010; Lau et al. 2021; Bergström et al. 2022, 2026). Given the broad boreal gradients represented here, these trajectories are likely relevant beyond Fennoscandia, underscoring the need for continued long‐term monitoring that links changing atmospheric forcing to lake chemistry and seasonal biological responses.

## Author Contributions

**Aleksey Paltsev:** conceptualization, investigation, writing – original draft, methodology, validation, visualization, writing – review and editing, formal analysis, data curation. **Erik Geibrink:** methodology, writing – review and editing, data curation, project administration, investigation. **Ann‐Kristin Bergström:** conceptualization, investigation, funding acquisition, writing – original draft, methodology, writing – review and editing, supervision, project administration, validation, visualization, formal analysis, resources. **Pirkko Kortelainen:** conceptualization, writing – review and editing, data curation, validation. **Dag O. Hessen:** conceptualization, investigation, writing – review and editing, data curation, methodology, visualization, supervision. **Jussi Vuorenmaa:** conceptualization, writing – review and editing, data curation, validation. **Kristiina Vuorio:** conceptualization, writing – review and editing, data curation, validation. **Anders Jonsson:** writing – review and editing, data curation, methodology, investigation, project administration. **Stina Drakare:** conceptualization, investigation, writing – review and editing, methodology, validation, data curation, supervision. **Heleen A. de Wit:** data curation, writing – review and editing. **Peter D. F. Isles:** conceptualization, writing – review and editing, methodology, validation. **Tobias Vrede:** conceptualization, writing – original draft, data curation, formal analysis, validation, writing – review and editing, methodology, supervision. **Irena F. Creed:** conceptualization, investigation, funding acquisition, writing – original draft, writing – review and editing, methodology, validation, formal analysis, supervision, resources. **Danny C. P. Lau:** conceptualization, investigation, writing – original draft, writing – review and editing, methodology, validation, formal analysis, data curation, funding acquisition, resources, visualization. **Kimmo K. Kahilainen:** conceptualization, investigation, writing – review and editing, methodology, data curation.

## Funding

This study was supported by the following funding sources: Grants from the Swedish Research Council (VR; no. 2020‐03224) were led by Ann‐Kristin Bergström. Aleksey Paltsev was supported by the Carl Trygger foundation (CTS 21:1145) to Ann‐Kristin Bergström and Irena F. Creed. Natural Sciences and Engineering Research Council of Canada (NSERC) Discovery Grant (2024‐04857) to Irena F. Creed. Grants from the Swedish Research Council for the Environment, Agricultural Sciences and Spatial Planning (FORMAS; nos. 2021‐01062 and 2024‐02205) to Danny C. P. Lau.

## Conflicts of Interest

The authors declare no conflicts of interest.

## Supporting information


**Figure S1:** Lake density plots based on water DIN:TP ratio and TOC concentration of the lakes in individual subregions in (a) 1991–1995 and (b) 2015–2019. Contours and intensity of colors are bivariate nonparametric density surface fits indicating the density of data points: 100%, 67%, and 33% density of the lakes in each subregion. The vertical broken line indicates the TOC threshold above which lakes shift from clear‐water, nutrient‐limited conditions to brown, light‐limited conditions (Solomon et al. 2015). Horizontal broken lines indicate log DIN:TP thresholds of nutrient limitation regimes for phytoplankton: > 0.9 for P‐limitation, 0.5–0.9 for N and P co‐limitation, and < 0.5 for N‐limitation (Bergström 2010; Isles et al. 2018).
**Table S1:** Results of mixed linear models for the 1991–2019 time series of lake water chemistry and atmospheric variables (air temperature, precipitation, N deposition and precipitation) for each subregion of Fennoscandia. Lakes within each subregion were used as a random factor in the models. Negative (or positive) coefficient estimates of atmospheric variables indicate negative (or positive) effects on the water chemistry variables. *p*‐values smaller than 0.05 are boldfaced.
**Table S2:** *p*‐values of mixed linear models (MLM) for the 1991–2019 time series of lake water chemistry and atmospheric variables (air temperature, precipitation, N deposition and precipitation) for each subregion of Fennoscandia. Significant *p*‐values after Holm–Bonferroni corrections are boldfaced. See Table S1 for statistics of the random and fixed effects in each MLM.

## Data Availability

Raw data on water chemistry and Chl‐*a* from Swedish lakes are hosted at the Department of Aquatic Sciences and Assessment at the Swedish University of Agricultural Sciences. Raw data on water chemistry from Norwegian and Finnish lakes are hosted at Norwegian Institute for Water Research and the Finnish lakes at the Finnish Environment Institute, respectively. The raw data that support the findings of this study are openly available at Zenodo (https://doi.org/10.5281/zenodo.18664051). The code used to analyse the data is available at: https://github.com/AleseyPA/Global‐change‐reshapes‐northern‐lakes‐R‐script.git.
