# Supplementary material for: Global Change Reshapes Northern Lakes Towards Browner, More Nutrient‐Depleted and Nitrogen‐Limited Conditions With Contrasting Impacts on Phytoplankton Biomass
Source: Glob Chang Biol. 2026 Jul 22;32(7):e71008. doi: 10.1111/gcb.71008 (PMC13389825; doi:10.1111/gcb.71008)
Supplement: Supplementary file 1 — Figure S1: Lake density plots based on water DIN:TP ratio and TOC concentration of the lakes in individual subregions in (a) 1991–1995 and (b) 2015–2019. Contours and intensity of colors are bivariate nonparametric density surface fits indicating the density of data points: 100%, 67%, and 33% density of the lakes in each subregion. The vertical broken line indicates the TOC threshold above which lakes shift from clear‐water, nutrient‐limited conditions to brown, light‐limited conditions (Solomon et al. 2015). Horizontal broken lines indicate log DIN:TP thresholds of nutrient limitation regimes for phytoplankton: > 0.9 for P‐limitation, 0.5–0.9 for N and P co‐limitation, and < 0.5 for N‐limitation (Bergström 2010; Isles et al. 2018). Table S1: Results of mixed linear models for the 1991–2019 time series of lake water chemistry and atmospheric variables (air temperature, precipitation, N deposition and precipitation) for each subregion of Fennoscandia. Lakes within each subregion were used as a random factor in the models. Negative (or positive) coefficient estimates of atmospheric variables indicate negative (or positive) effects on the water chemistry variables. p‐values smaller than 0.05 are boldfaced. Table S2: p‐values of mixed linear models (MLM) for the 1991–2019 time series of lake water chemistry and atmospheric variables (air temperature, precipitation, N deposition and precipitation) for each subregion of Fennoscandia. Significant p‐values after Holm–Bonferroni corrections are boldfaced. See Table S1 for statistics of the random and fixed effects in each MLM. [file GCB-32-e71008-s001.docx]

**Supplementary Information**

**Global change reshapes northern lakes towards browner, more nutrient-depleted and nitrogen-limited conditions with contrasting impacts on phytoplankton biomass**

**Authors:** Ann-Kristin Bergström^1†^, Aleksey Paltsev^1^, Dag O. Hessen^2^, Pirkko Kortelainen^3^, Jussi Vuorenmaa^3^, Heleen A de Wit^2,4^, Danny C. P. Lau^5^, Tobias Vrede^5^, Kristiina Vuorio^3^_,_ Peter D. F. Isles^6^, Anders Jonsson^1^, Erik Geibrink^1^, Kimmo K. Kahilainen^7^, Stina Drakare^5^, Irena F. Creed^8^

^1^ Department of Ecology, Environment and Geoscience, Umeå University, Umeå, Sweden

^2^ Centre of Biogeochemistry in the Anthropocene and Department of Bioscience, University of Oslo, Oslo, Norway

^3^ Finnish Environment Institute (SYKE), Helsinki, Finland

^4^ Norwegian Institute for Water Research, Oslo, Norway

^5^ Department of Aquatic Sciences and Assessment, Swedish University of Agricultural Sciences, Uppsala, Sweden

^6^ Watershed Management Division, Vermont Department of Environmental Conservation, Montpelier, Vermont, U.S.A.

^7^ Lammi Biological Station, University of Helsinki, Helsinki, Finland

^8^ Department of Physical and Environmental Sciences, University of Toronto, Toronto, Ontario, Canada

**Table S1.** Results of mixed linear models for the 1991-2019 time series of lake water chemistry and atmospheric variables (air temperature, precipitation, N deposition and precipitation) for each subregion of Fennoscandia. Lakes within each subregion were used as a random factor in the models. Negative (or positive) coefficient estimates of atmospheric variables indicate negative (or positive) effects on the water chemistry variables. *P* values smaller than 0.05 are boldfaced.

|  |  |  | Log10(TOC) | | Log10(TP) |  | Log10(DIN) | | Log10(DIN:TP) | | Log10(TOC:TP) | |
| --- | --- | --- | --- | --- | --- | --- | --- | --- | --- | --- | --- | --- |
| Subregion | Random | Fixed | Estimate | *P* | Estimate | *P* | Estimate | *P* | Estimate | *P* | Estimate | *P* |
| **Finland north** | Lake |  | 0.065 | **0.050** | 0.044 | 0.059 | 0.069 | 0.204 | 0.064 | 0.094 | 0.012 | 0.066 |
|  |  | Intercept | -2.088 | **<0.001** | 0.551 | 0.293 | -0.539 | 0.593 | -0.314 | 0.753 | 0.530 | 0.291 |
|  |  | Temperature | -0.002 | 0.771 | -0.016 | 0.214 | -0.077 | **0.004** | -0.097 | **<0.001** | -0.020 | 0.134 |
|  |  | Log10(Precipitation) | 0.953 | **<0.001** | -0.128 | 0.484 | 0.399 | 0.268 | 0.378 | 0.297 | 0.955 | **<0.001** |
|  |  | Log10(N deposition) | 0.237 | **0.040** | 0.295 | 0.052 | 0.254 | 0.454 | -0.020 | 0.951 | 0.096 | 0.545 |
|  |  | Log10(S deposition) | -0.259 | **<0.001** | -0.002 | 0.982 | 0.024 | 0.886 | 0.096 | 0.565 | -0.230 | **0.007** |
| **Finland south** | Lake |  | 0.039 | **0.035** | 0.048 | **0.041** | 0.067 | **0.047** | 0.132 | **0.042** | 0.024 | **0.044** |
|  |  | Intercept | 0.471 | **0.042** | -0.715 | 0.228 | -1.139 | 0.287 | -0.831 | 0.462 | 3.282 | **<0.001** |
|  |  | Temperature | -0.005 | 0.393 | 0.015 | 0.354 | -0.024 | 0.401 | -0.025 | 0.408 | -0.005 | 0.737 |
|  |  | Log10(Precipitation) | 0.138 | 0.093 | 0.037 | 0.859 | 0.325 | 0.411 | 0.356 | 0.392 | 0.142 | 0.474 |
|  |  | Log10(N deposition) | 0.214 | 0.052 | 0.508 | 0.113 | 0.745 | 0.155 | 0.365 | 0.509 | -0.058 | 0.825 |
|  |  | Log10(S deposition) | -0.300 | **<0.001** | -0.009 | 0.944 | -0.022 | 0.907 | 0.039 | 0.843 | -0.263 | **0.006** |
| **Sweden northeast** | Lake |  | 0.016 | **0.009** | 0.025 | **0.012** | 0.052 | **0.011** | 0.068 | **0.011** | 0.008 | **0.019** |
|  |  | Intercept | -0.399 | **0.032** | 0.339 | 0.510 | -0.302 | 0.591 | -1.261 | 0.053 | 1.414 | **<0.001** |
|  |  | Temperature | -0.012 | **0.017** | 0.005 | 0.630 | -0.009 | 0.524 | -0.007 | 0.673 | -0.012 | 0.237 |
|  |  | Log10(Precipitation) | 0.534 | **<0.001** | -0.099 | 0.527 | 0.234 | 0.222 | 0.474 | **0.032** | 0.768 | **<0.001** |
|  |  | Log10(N deposition) | 0.122 | 0.140 | 0.496 | **0.010** | 0.522 | **0.037** | 0.513 | 0.076 | 0.072 | 0.665 |
|  |  | Log10(S deposition) | -0.161 | **<0.001** | -0.162 | 0.077 | -0.037 | 0.686 | -0.195 | 0.063 | -0.311 | **<0.001** |
| **Sweden northwest** | Lake |  | 0.125 | **0.011** | 0.027 | **0.027** | 0.048 | **0.012** | 0.042 | **0.014** | 0.059 | **0.035** |
|  |  | Intercept | -0.011 | 0.972 | 0.071 | 0.918 | -0.002 | 0.998 | -0.813 | 0.255 | 1.314 | **0.032** |
|  |  | Temperature | -0.004 | 0.662 | 0.010 | 0.477 | -0.014 | 0.382 | -0.004 | 0.804 | 0.027 | 0.083 |
|  |  | Log10(Precipitation) | 0.201 | **0.046** | -0.175 | 0.452 | -0.117 | 0.580 | 0.564 | **0.021** | 0.842 | **<0.001** |
|  |  | Log10(N deposition) | 0.084 | 0.444 | 0.596 | **<0.001** | 0.514 | **0.020** | -0.114 | 0.648 | -0.195 | 0.370 |
|  |  | Log10(S deposition) | -0.064 | 0.175 | -0.073 | 0.455 | 0.277 | **0.005** | 0.117 | 0.295 | -0.317 | **<0.001** |
| **Sweden southeast** | Lake |  | 0.018 | **0.010** | 0.046 | **0.009** | 0.054 | **0.011** | 0.088 | **0.011** | 0.026 | **0.010** |
|  |  | Intercept | -0.189 | 0.471 | 1.106 | **0.009** | 0.794 | 0.261 | -0.310 | 0.700 | 1.442 | **0.001** |
|  |  | Temperature | 0.006 | 0.354 | -0.008 | 0.440 | -0.047 | **0.007** | -0.047 | **0.020** | 0.008 | 0.480 |
|  |  | Log10(Precipitation) | 0.339 | **<0.001** | -0.126 | 0.350 | -0.254 | 0.308 | -0.056 | 0.843 | 0.512 | **0.001** |
|  |  | Log10(N deposition) | 0.230 | 0.072 | 0.196 | 0.320 | 0.812 | **0.019** | 0.908 | **0.022** | 0.314 | 0.153 |
|  |  | Log10(S deposition) | -0.212 | **<0.001** | -0.124 | 0.072 | -0.068 | 0.512 | -0.198 | 0.095 | -0.338 | **<0.001** |
| **Sweden southwest** | Lake |  | 0.046 | **<0.001** | 0.040 | **<0.001** | 0.045 | **<0.001** | 0.055 | **<0.001** | 0.015 | **<0.001** |
|  |  | Intercept | -0.157 | 0.478 | 0.771 | 0.055 | -0.088 | 0.865 | -0.455 | 0.435 | 2.199 | **<0.001** |
|  |  | Temperature | -0.012 | **0.028** | -0.017 | 0.069 | -0.031 | **0.022** | -0.026 | 0.091 | -0.004 | 0.608 |
|  |  | Log10(Precipitation) | 0.665 | **<0.001** | -0.345 | **0.008** | 0.416 | **0.025** | 0.636 | **0.003** | 0.863 | **<0.001** |
|  |  | Log10(N deposition) | -0.141 | 0.170 | 0.632 | **<0.001** | 0.285 | 0.227 | -0.027 | 0.918 | -0.468 | **0.002** |
|  |  | Log10(S deposition) | -0.121 | **<0.001** | -0.144 | **0.020** | 0.056 | 0.431 | 0.001 | 0.988 | -0.171 | **<0.001** |
| **Norway north** | Lake |  | 0.202 | **<0.001** | 0.028 | **<0.001** | 0.125 | **<0.001** | 0.221 | **<0.001** | 0.071 | **<0.001** |
|  |  | Intercept | 0.161 | 0.395 | 0.514 | 0.084 | 1.313 | **<0.001** | 1.852 | **<0.001** | 3.510 | **<0.001** |
|  |  | Temperature | 0.014 | **<0.001** | -0.003 | 0.749 | -0.024 | **0.002** | -0.010 | 0.295 | 0.037 | **<0.001** |
|  |  | Log10(Precipitation) | 0.096 | 0.095 | -0.252 | **0.023** | -0.414 | **<0.001** | -0.475 | **<0.001** | -0.089 | 0.356 |
|  |  | Log10(N deposition) | -0.052 | 0.195 | 0.346 | **<0.001** | 0.489 | **<0.001** | 0.259 | **0.007** | -0.180 | **0.009** |
|  |  | Log10(S deposition) | -0.074 | **<0.001** | -0.042 | 0.382 | 0.162 | **<0.001** | 0.168 | **<0.001** | -0.095 | **0.005** |
| **Norway southeast** | Lake |  | 0.135 | **0.016** | 0.053 | **0.023** | 0.069 | **0.018** | 0.135 | **0.015** | 0.034 | **0.026** |
|  |  | Intercept | -1.220 | **<0.001** | 0.515 | 0.480 | 2.666 | **<0.001** | 2.457 | **<0.001** | 1.152 | **0.006** |
|  |  | Temperature | -0.012 | 0.084 | 0.009 | 0.567 | -0.011 | 0.293 | -0.013 | 0.350 | 0.000 | 0.995 |
|  |  | Log10(Precipitation) | 0.603 | **<0.001** | -0.061 | 0.748 | -0.598 | **<0.001** | -0.582 | **<0.001** | 0.522 | **<0.001** |
|  |  | Log10(N deposition) | 0.387 | **<0.001** | 0.138 | 0.557 | 0.245 | 0.088 | 0.184 | 0.308 | 0.446 | **0.003** |
|  |  | Log10(S deposition) | -0.334 | **<0.001** | -0.038 | 0.660 | 0.186 | **<0.001** | 0.180 | **0.002** | -0.365 | **<0.001** |
| **Norway southwest** | Lake |  | 0.170 | **<0.001** | 0.090 | **0.002** | 0.086 | **0.002** | 0.166 | **<0.001** | 0.060 | **<0.001** |
|  |  | Intercept | -0.781 | **0.024** | -1.624 | **0.027** | 2.201 | **<0.001** | 2.907 | **<0.001** | 2.644 | **<0.001** |
|  |  | Temperature | 0.015 | 0.069 | -0.042 | **0.003** | -0.024 | **0.011** | 0.003 | 0.823 | 0.048 | **<0.001** |
|  |  | Log10(Precipitation) | 0.153 | 0.127 | 0.349 | **0.050** | -0.391 | **<0.001** | -0.515 | **0.001** | -0.019 | 0.889 |
|  |  | Log10(N deposition) | 0.494 | **<0.001** | 0.610 | **<0.001** | 0.224 | **0.027** | -0.105 | 0.453 | 0.172 | 0.154 |
|  |  | Log10(S deposition) | -0.402 | **<0.001** | -0.161 | **0.022** | 0.266 | **<0.001** | 0.376 | **<0.001** | -0.282 | **<0.001** |

**Table S2.** *P* values of mixed linear models (MLM) for the 1991-2019 time series of lake water chemistry and atmospheric variables (air temperature, precipitation, N deposition and precipitation) for each subregion of Fennoscandia. Significant *P* values after Holm–Bonferroni corrections are boldfaced. See Table S1 for statistics of the random and fixed effects in each MLM.

|  | Model *P* | | | | |
| --- | --- | --- | --- | --- | --- |
| Subregion | Log10(TOC) | Log10(DIN) | Log10(TP) | Log10(DIN:TP) | Log10(TOC:TP) |
| Finland north | **<0.0001** | **0.0014** | 0.0492 | **0.0001** | **<0.0001** |
| Finland south | **<0.0001** | **0.0028** | 0.0638 | 0.0684 | **<0.0001** |
| Norway north | **<0.0001** | **<0.0001** | **0.0003** | **<0.0001** | **<0.0001** |
| Norway southeast | **<0.0001** | **<0.0001** | 0.9376 | **<0.0001** | **<0.0001** |
| Norway southwest | **<0.0001** | **<0.0001** | **0.0014** | **<0.0001** | **<0.0001** |
| Sweden northeast | **<0.0001** | **0.0002** | 0.1330 | 0.0122 | **<0.0001** |
| Sweden northwest | 0.1621 | **<0.0001** | **0.0004** | 0.0964 | **<0.0001** |
| Sweden southeast | **<0.0001** | **<0.0001** | 0.2415 | 0.0067 | **<0.0001** |
| Sweden southwest | **<0.0001** | **<0.0001** | **0.0027** | 0.0065 | **<0.0001** |


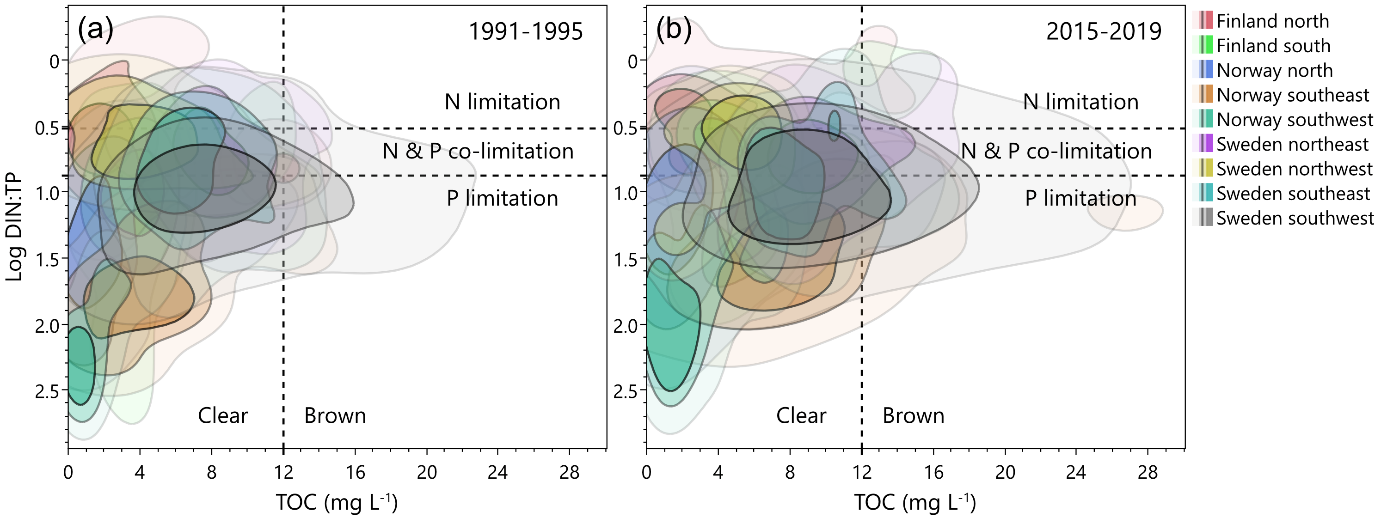


Figure S1. Lake density plots based on water DIN:TP ratio and TOC concentration of the lakes in individual subregions in (a) 1991-1995 and (b) 2015-2019. Contours and intensity of colors are bivariate nonparametric density surface fits indicating the density of data points: 100%, 67%, and 33% density of the lakes in each subregion. The vertical broken line indicates the TOC threshold above which lakes shift from clear-water, nutrient-limited conditions to brown, light-limited conditions (Solomon et al. 2015). Horizontal broken lines indicate log DIN:TP thresholds of nutrient limitation regimes for phytoplankton: >0.9 for P-limitation, 0.5-0.9 for N and P co-limitation, and <0.5 for N-limitation (Bergström 2010; Isles et al., 2018).
